# Granulosa Cells Improved Mare Oocyte Cytoplasmic Maturation by Providing Collagens

**DOI:** 10.3389/fcell.2022.914735

**Published:** 2022-06-30

**Authors:** Xinyuan Zhu, Shanshan Zhao, Shibo Xu, Dongyu Zhang, Minghui Zhu, Qingjie Pan, Jiaojiao Huang

**Affiliations:** College of Animal Science and Technology, Qingdao Agricultural University, Qingdao, China

**Keywords:** mare, oocyte, cytoplasm maturation, granulosa cells, BMP15, collagens

## Abstract

Assisted reproductive technology has important clinical applications and commercial values in the horse industry. However, this approach is limited largely by the low efficiency of oocyte *in vitro* maturation (IVM), especially cytoplasmic maturation. To improve the efficiency of mare oocyte IVM, we evaluated the effects of co-culture with cumulus–oocyte complexes (COCs) and granulosa cells (GCs) from follicles with small (<15 mm) and large diameters (>35 mm). Our results showed that oocyte nucleus maturation was not significantly improved by co-culturing with GCs. Interestingly, the cytoplasmic maturation of oocytes, defined by the distribution of cortical granules and mitochondria, as well as reactive oxygen species (ROS) levels, improved dramatically by co-culture with GCs, especially those derived from small follicles. Moreover, GCs promoted cumulus cell expansion by upregulating the expression of BMP15 in oocytes. To determine the mechanism underlying the effects of GCs, the transcriptomes of GCs from large and small follicles were compared. Expression levels of *COL1A2*, *COL6A1*, and *COL6A2* were significantly higher in GCs from small follicles than in those from large follicles. These three genes were enriched in the extracellular matrix proteins-receptor interaction pathway and were involved in the regulation of collagens. Taken together, our results suggest that co-culture with GCs is beneficial to oocyte cytoplasmic maturation, and the increased expression of *COL1A2*, *COL6A1*, and *COL6A2* improve the mare oocyte IVM system *via* the regulation of collagen.

## Introduction

Horses are one of the few species other than humans in which assisted reproductive technology has significant clinical applications and is beneficial to the commercial value of the animal (Lewis et al., 2020). Furthermore, the horse is used as a natural model for research on reproductive aging-induced oocyte aneuploidy as well as comparative reproductive biology (Rizzo et al., 2020; Benammar et al., 2021). However, due to the low efficiency of oocyte *in vitro* maturation (IVM), clinical translation of assisted reproductive technologies in horses remains limited. Despite the success of the IVM of mammalian oocytes in several species, including pig, mouse, sheep, and cow (Dadashpour Davachi et al., 2017; Wang et al., 2018; Yang et al., 2020; Ding et al., 2021), this procedure has not been optimized for horses owing to the limited supply of oocytes and the specific physiology of the species.

The mammalian ovary contains a huge number of oocytes at various stages of development. *In vitro* growth gives these oocytes meiotic and developmental competence, resulting in a consistent supply of mature oocytes for reproductive medicine as well as livestock and endangered species breeding (Yamochi et al., 2021). Somatic cells inside the follicular environment (granulosa and cumulus cells) play a significant role in the formation of oocytes and female reproduction (Del Collado et al., 2018). Granulosa cells (GCs) are critical cells in follicles, with important roles in follicular development and atresia *via* the secretion of growth factors and gap junctional communications (Kidder and Vanderhyden, 2010; Matsuda et al., 2012; Jaffe and Egbert, 2017).

Horse cumulus–oocyte complexes (COC) are firmly connected to the follicular wall (Merlo et al., 2018). When aspirating COCs, 63% have cumulus cells with only the corona radiata present in their morphology (Hinrichs, 1991). GCs not only play an important role in nuclear maturation by responding to gonadotropins during folliculogenesis but also regulate cytoplasmic maturation (Russell, 1998; Mori et al., 2000). The effects of GCs on the follicular development and oocyte maturation *in vitro* have been studied extensively to better simulate the physiologic context of the follicular growth *in vivo*. The quality of oocytes is critical for later embryonic development, and growth factors secreted by GCs are important nutrients for the oocyte maturation *in vivo* and are lacking in typical *in vitro* maturation systems (De Prada et al., 2009). When co-cultured with analogous GCs, murine (Gilchrist et al., 2004), porcine (Hickey et al., 2005), buffalo (Zhang et al., 2022), and bovine oocytes (Gilchrist et al., 2003), maturation is substantially enhanced. However, to our knowledge, little is known about the effects of GCs on the IVM of mare oocytes.

Hence, in the present study, we investigated the effect of co-culturing GCs with the mare oocyte maturation *in vitro.* Our findings indicated that the mare oocyte nuclear maturation is not significantly improved by co-culture with GCs. We further compared the effects of GCs originating from the follicles of different sizes on the mare oocyte cytoplasmic maturation, as measured by the distribution of cortical granules and mitochondria as well as the level of reactive oxygen species (ROS). The underlining mechanism was investigated by RNA-seq of GCs, which revealed that elevated levels of collagen-encoding genes (*COL1A2*, *COL6A1*, and *COL6A2*) provide a favorable milieu for the mare oocyte IVM.

## Materials and Methods

### Ethics Statement

The ethical committee of Qingdao Agricultural University evaluated and approved all of the procedures reported in the study (Agreement No. DEC2019-18).

### Media and Reagents

Unless otherwise noted, all compounds were purchased from Sigma (St. Louis, MO, United States). A 0.22-mm filter was used to filter all of the following solutions and materials.

TCM199 (Gibco BRL, San Francisco, CA, United States) supplemented with 1% (v/v) penicillin/streptomycin, 10% fetal bovine serum (BSA, Hyclone, Logan, UT, United States), 0.01 IU/ml luteinizing hormone, 0.01 IU/ml follicle-stimulating hormone, 1 μg/ml 17β-estrogen, 50 ng/ml epidermal growth factor, 0.11 mg/ml sodium pyruvate, and 200 ng/ml insulin-like growth factor-1 were used for the oocyte IVM.

### Collection of Granulosa Cells and Co-Cultured With Cumulus–Oocyte Complexes

Ovaries were collected from a horse slaughterhouse in Jimo District, Qingdao, Shandong Province, China; stored in saline; and transported to the laboratory at 37°C. Follicular fluids were aspirated from large follicles (diameter >35 mm) and small follicles (diameter <15 mm), with an 18-gauge needle attached to a disposable 10 ml syringe. After examination under a stereomicroscope to locate and collect COCs, the remaining liquid was centrifuged (300 × g, 10 min) and the GCs were immediately resuspended in the IVM medium. GCs were plated onto four-well cell culture plates (Nunc, Roskilde, and Denmark) at a density of 1 × 10^6^ cells per well.

Follicular contents were obtained using the scraping method according to previously described methods (Hinrichs et al., 2005). COCs in follicular fluid were gravity settled at 38.5°C. IVM was limited to COCs with numerous layers of intact cumulus cells and a homogeneous ooplasm. A group of 20–40 COCs were plated onto four-well cell culture plates containing 500 μl of the IVM medium and 350 μl of mineral oil in each well after washing three times in the IVM medium. Then, COCs were randomly assigned to the following experimental groups: COCs cultured in the IVM medium (CONTROL), COCs cultured in the IVM medium containing 1 × 10^6^/well small follicle–derived GCs (SFGC + O), and COCs cultured in the IVM medium containing 1×10^6^/well large follicle–derived GCs (LFGC + O). The COCs were cultured at 38.5°C and 5% CO_2_ (100% humidity). After culturing for 4 h, a portion of COCs was used to remove cumulus cells by vortexing the HEPES-buffered Tyrode medium containing 0.1% hyaluronidase and 0.01% polyvinyl alcohol (PVA) for 4 min, followed by analyses of the meiotic stages of oocytes. After culturing for 36 h, the cumulus cells were removed from the remaining COCs, followed by nuclear maturation. Only MII oocytes with an extruded first polar body (PB) were used for further analyses.

### Evaluation of Cortical Granules Distribution

MII oocytes were first fixed with 4% (v/v) paraformaldehyde dissolved in phosphate-buffered saline (PBS). After permeabilizing with 1% (v/v) tritonX-100 dissolved in PBS (30 min), the oocytes were stained for 30 min with 100 mg/ml fluorescein isothiocyanate–labeled peanut agglutinin (FITC-PNA, Vector Laboratories, Burlingame, CA). Subsequently, the stained oocytes were placed on glass slides and examined using a confocal laser scanning microscope (Zeiss, Oberkochen, Germany). The cortical granule distribution was categorized as follows: peripheral (cortical granules were adjacent to the plasma membrane, indicating cytoplasmic maturation), cortical (cortical granules were localized in the cortical area of oocytes, indicating partial maturation), homogeneous (cortical granules were scattered throughout the cytoplasm, indicating a lack of cytoplasmic maturation), and abnormal distribution (cortical granules had an irregular distribution, indicating a poor quality or deterioration), according to a previous study (Velilla et al., 2004).

### Localization and Distribution of Mitochondria in the Oocyte

MII oocytes were stained for mitochondria by MitoTracker Red CMXRos. According to the manufacturer’s recommendation, a stock solution at a concentration of 1 mM was produced in dimethyl sulfoxide and stored at −20°C (Irvine Scientific, Santa Ana, CA, United States). Oocytes were stained in the modified human tubular fluid containing 0.5 mM MitoTracker Red CMXRos for 30 min at 37°C. The samples were then fixed in PBS containing 2% (w/v) paraformaldehyde for 10 min at 37°C. To detect DNA, the oocytes were treated to 10 mg/ml Hoechst 33342 for 10 min. Then, they were mounted on glass slides and examined using laser scanning confocal microscopy.

### Measurement of Intracellular Reactive Oxygen Species Levels

Oocytes from each group were incubated (in the dark) in the TCM199 medium supplemented with 100 M 2, 7-dichlorodihydrofluorescein diacetate (DCFH-DA, Biyuntian, China) for 20 min at 37°C, and then washed three times in TCM199 medium containing 3 mg/ml bovine serum albumin according to the manufacturer’s instructions. Subsequently, the stained oocytes were put on glass slides and examined using a confocal laser scanning microscope. After subtracting the background value, the recorded fluorescence intensities were measured using ImageJ.

### Assessment of Cumulus Cell Expansion

The degree of cumulus cell expansion was assessed after IVM, according to previously described methods (Vanderhyden et al., 1990). In brief, a lack of response was assigned a score of 0, the minimum observable response was assigned a score of 1, the observed expansion of outer cumulus cell layers was assigned a score of 2, the expansion of all cumulus cell layers except the corona radiate was assigned a score of 3, and the expansion of all cumulus cell layers including the corona radiate was assigned a score of 4 (Vanderhyden et al., 1990).

### RNA-Seq and Analysis

GCs from large follicles (LGFC) and small follicles (SGFC) were collected using the above method and immediately frozen at −80°C. Total RNA was isolated and purified from GCs using the TRIzol reagent (Invitrogen, Carlsbad, CA, United States) according to the manufacturer’s instructions. The cDNA library construction, sequencing, and transcriptome data analysis were conducted by LC-Bio Technology CO., Ltd (LC-Bio, Hangzhou, China). Fold change (FC) ≥ 2 or FC ≤ 0.5 (i.e., absolute value of log_2_FC ≥ 1) were used as thresholds for the detection of differentially expressed genes (DEGs), and *p* < 0.05 was employed to screen for Gene Ontology and KEGG pathway enrichment.

### Fluorescence Immunoassay

After samples were rinsed in PBS, the oocytes were fixed in 4% paraformaldehyde in PBS for 15 min and permeabilized in 1% TritonX-100 in PBS for 30 min. Then the oocytes were blocked in 5% BSA in PBS for 1 h at room temperature. Then, the samples were incubated overnight at 4°C with primary antibodies against BMP15 (1:1000, ABclonal Technology Co., Ltd., Woburn, MA, United States) according to the manufacturer’s instructions. After extensive washing with PBS, the samples were incubated with FITC rabbit anti-goat IgG (1:1000, ABclonal) for 1 h at room temperature, according to the manufacturer’s instructions. Then the oocytes were treated with 10 mg/ml Hoechst 33342 for 10 min to detect DNA. They were mounted on glass slides and examined using laser scanning confocal microscopy. For comparisons, the exposure and image capture settings were kept constant, and all images were compiled without any contrast or brightness adjustments.

### Quantitative Polymerase Chain Reaction Analysis

Total RNAs of GCs from LGFC and SGFC were isolated and purified following the aforementioned methods. TransScript-Uni Cell to cDNA Synthesis SuperMix (TransGen Biotech, Beijing, China) was used to obtain cDNA according to the manufacturer’s instructions. Then, cDNA was quantified by a quantitative polymerase chain reaction (Q-PCR) using a TransStart Tip Green qPCR SuperMix Kit (TransGen Biotech) and a real-time detection system (Bio-Rad, Hercules, CA, United States) under standard conditions. At least three replicates were performed for this assay, and the 2^−∆∆Ct^ method was used to determine the relative mRNA expression levels. For normalization, *GAPDH* was used as a reference gene (da Silveira et al., 2015). Primer information is detailed in Supplementary Table S1.

### Statistical Analyses

Every experiment was carried out at least three times. Differences in oocyte nuclear maturation, cortical granule distribution, distributions of mitochondria, and ROS levels were evaluated by chi-squared tests. Differences in the relative expression levels of target genes were evaluated based on corrected quantitative fluorescence levels for each sample by one-way analysis of variance using SAS 6.12. (SAS Institute, Cary, NC, United States). *p* < 0.05 (*) or *p* < 0.01 (**) indicated significance.

## Results

### Mare Oocyte Nucleus Maturation Was Not Affected by Co-Culturing With Granulosa Cells

**FIGURE 1:**
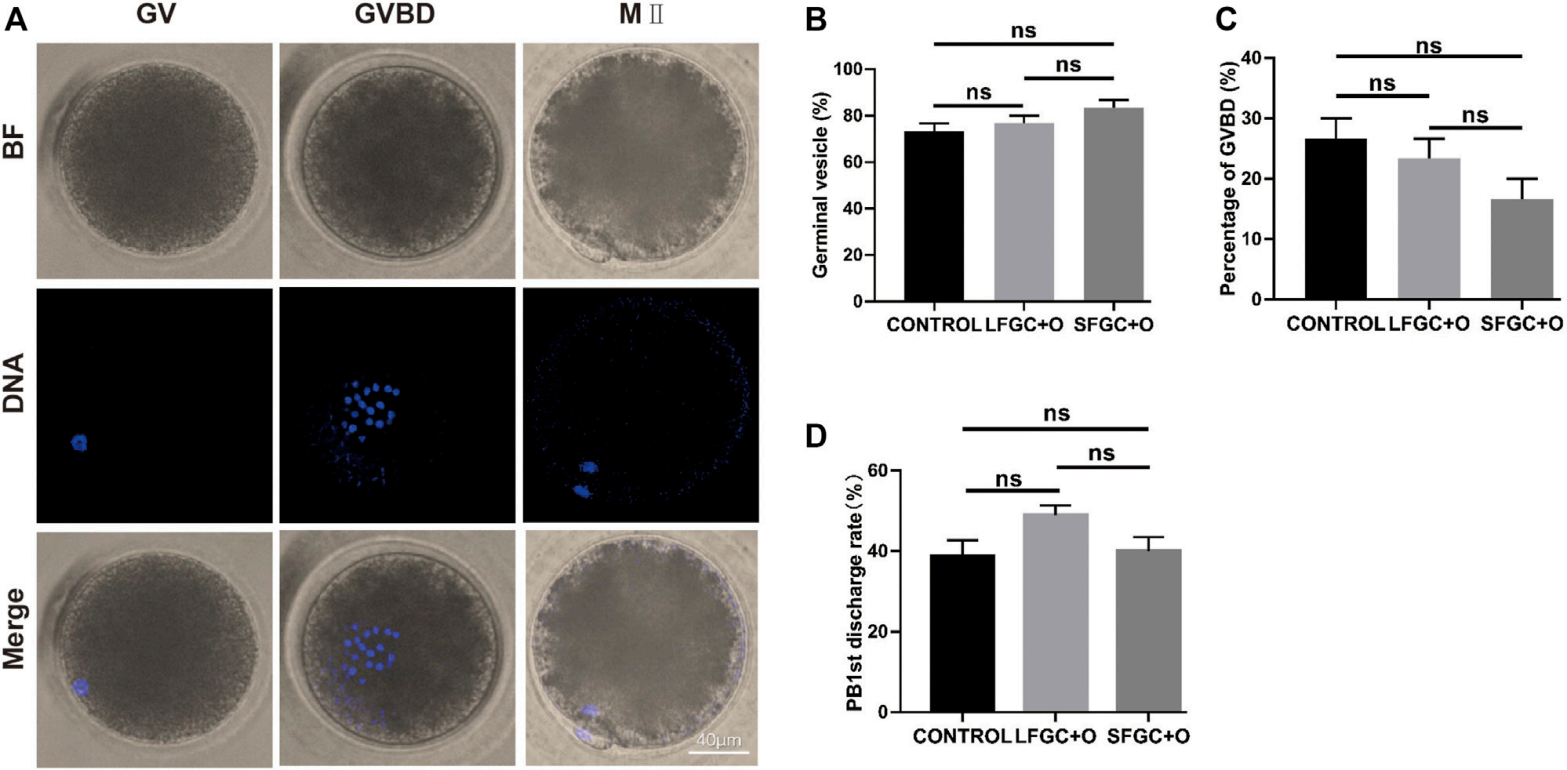
Effects of GC co-culture on mare oocyte *in vitro* nuclear maturation. **(A)** Nuclear staining of oocytes in GV, GVBD, and MII stages. Scale bar = 40 µm. **(B)** Percentages of GV stage mare oocytes derived from the CONTROL, LFGC + O, and SFGC + O groups after 4 h of culture *in vitro* (30 oocytes per group). **(C)** Percentages of GVBD stage mare oocytes derived from the CONTROL, LFGC + O, and SFGC + O groups after 4 h of culture *in vitro* (30 oocytes per group). **(D)** Percentages of MII stage mare oocytes derived from the CONTROL, LFGC + O, and SFGC + O groups after 36 h of culture *in vitro* (76 oocytes per group). Data are shown as means ± SEM.

To verify the effect of GCs on mare oocyte development, COCs were cultured in the IVM medium only (CONTROL), medium containing 1 × 10^6^/well small follicle–derived GCs (SFGC + O), or medium containing 1 × 10^6^/well large follicle–derived GCs (LFGC + O), as described in the Materials and Methods section. After culturing for 4 h and 36 h *in vitro*, the oocytes were collected to evaluate meiotic stages, as shown in Figure 1A. As determined by Hoechst 33342 staining, there were no significant differences in the oocyte nuclear morphology among the three groups (Figure 1B). After culturing for 4 h, the germinal vesicle (GV) rates were 73.33, 76.67, and 83.33% in the control, LFGC, and SFGC groups (*p* > 0.05). The rates of germinal vesicle breakdown (GVBD) were 26.67, 23.33, and 16.67% in the control, LFGC, and SFGC groups (*p* > 0.05) (Figure 1C; Supplementary Table S2). After culturing for 36 h, the MII rates were 26.67, 23.33, and 16.67% in the control, LFGC, and SFGC groups (*p* > 0.05) (Figure 1C; Supplementary Table S2). After culturing for 36 h, the rates of MII were 38.83, 40, and 49% in the control, LFGC, and SFGC groups (*p* > 0.05) (Supplementary Table S3). These findings revealed that co-culture mare oocytes with GCs from follicles of various diameters did not increase nuclear maturation.

### Effect of Granulosa Cells on Cortical Granules Distribution in Mare MII Oocytes

**FIGURE 2:**
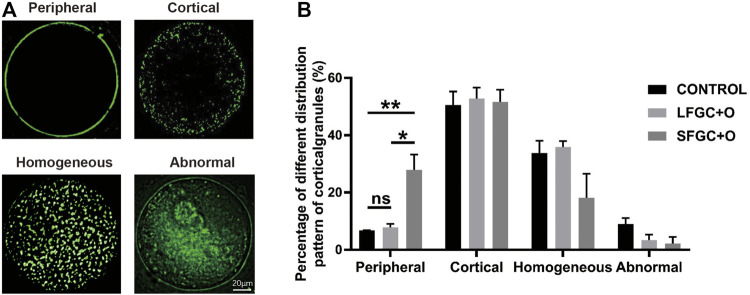
Effects of GC co-culture on the cortical granule distribution of mare MII oocytes. **(A)** Cortical granule staining with RNA-FITC in mare oocytes after *in vitro* maturation was detected by confocal microscopy. Scale bar = 20 µm. **(B)** Percentages of distributions classified as peripheral, cortical, homogeneous, and abnormal for cortical granules in mare MII oocytes derived from the CONTROL, LFGC-O, and SFGC-O groups (59 oocytes per group). Data are shown as means ± SEM. *p < 0.05 and **p < 0.01.

To determine whether co-culture with GCs affects the cytoplasmic maturation of mare oocytes, the distribution of cortical granules in oocytes was analyzed after IVM. The cortical granule distribution was classified as peripheral, cortical, homogeneous, and abnormal (Figure 2A). We found that co-culture with GCs significantly increased the frequency of the peripheral cortical granule distribution (indicating cytoplasmic maturation) compared with that in the control group, for both the SFGC group (27.93 ± 0.929 vs. 6.77 ± 0.115%, *p* < 0.01) and the LFGC group (7.9 ± 2.078 vs. 6.77 ± 0.115%, *p* < 0.05) (Figure 2B; Supplementary Table S4). These findings revealed that co-culturing mare oocytes with GCs from follicles of various diameters improved cytoplasmic maturation by regulating the distribution of cortical granules.

### Effect of Granulosa Cells on Mitochondria Distribution in Mare MII Oocytes

**FIGURE 3:**
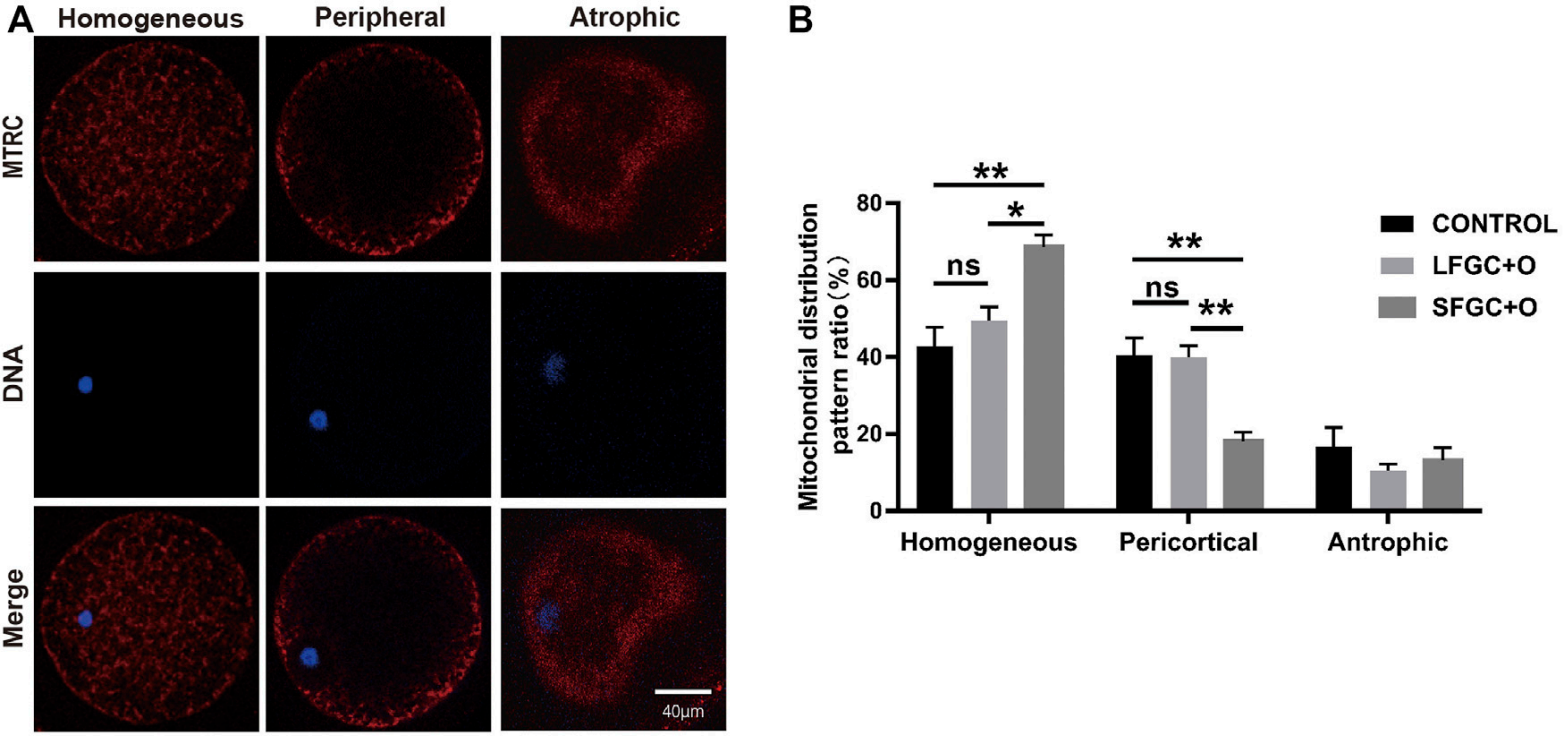
Effects of GC co-culture on mitochondrial distributions in mare MII oocytes. **(A)** Mitochondria stained with MitoTracker Red in mare MII oocytes after *in vitro* maturation were detected using confocal microscopy. Scale bar = 20 µm. **(B)** Percentages of homogeneous, peripheral, and atrophic distributions of mitochondria in mare MII oocytes derived from the CONTROL, LFGC-O, and SFGC-O groups (76 oocytes per group). Data are shown as means ± SEM. *p < 0.05 and **p < 0.01.

The distribution of mitochondria was visualized by MitoTracker Red CMXRos staining. Three mitochondrial distribution patterns in mare MII oocytes were identified, that is, homogeneous, peripheral, and atrophic (Figure 3A). Our results showed that the co-culture with GCs affects the distribution of mitochondria in MII oocytes. The homogeneous distribution of mitochondria was the most common pattern in MII oocytes of the three groups. The frequency of the homogeneous distribution was 67.67% ± 2.591% for SFGC-treated MII oocytes, which was significantly higher than those in the LFGC group (49.5 ± 3.585%, *p* < 0.05) and the control group (42.83 ± 4.963%, *p* < 0.01). Additionally, 14 ± 1.951% of SFGC-treated MII oocytes exhibited the peripheral distribution, which was significantly lower than the frequencies for the LFGC group (40 ± 2.887%, *p* < 0.01) and the control group (40 ± 2.887%, *p* < 0.01). GC co-culture had no significant effect on the frequency of atrophic mitochondria in MII oocytes (Figure 3B; Supplementary Table S5). These findings revealed that co-culture with GCs from small follicles promoted cytoplasmic maturation by regulating the distribution of mitochondria.

### Granulosa Cells Co-Culture Decreased Reactive Oxygen Species Production in Mare MII Oocytes

**FIGURE 4:**
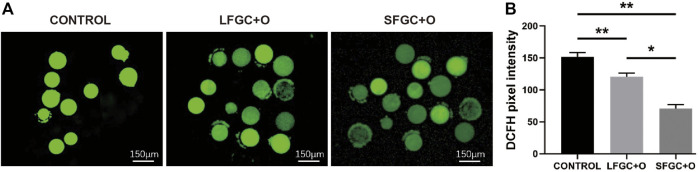
Effects of GC co-culture on ROS production in mare MII oocytes. **(A)** Representative fluorescence images were obtained from mare MII oocytes stained with DCFH-DA. Scale bar = 150 μm. **(B)** Relative fluorescence intensity of DCFH-DA in mare MII oocytes derived from the CONTROL, LFGC-O, and SFGC-O groups (30 oocytes per group). Average optical intensity was measured using ImageJ. Data are expressed as means ± SEM. *p < 0.05, **p < 0.01.

The ROS levels are correlated with the cytoplasmic quality of oocytes, which determines the subsequent developmental competency of oocytes and embryos. To determine the effect of GC co-culture on mare MII oocyte cytoplasmic maturation, ROS levels were detected by staining with DCFH-DA (Figure 4A). As shown in Figure 4A, ROS levels were significantly higher in control MII oocytes than after co-culture with GCs (*p* < 0.01). Notably, co-culture with SFGCs resulted in significantly lower ROS levels in MII oocytes than those in the LFGC-treated group (*p* < 0.05) (Figure 4B). These findings imply that co-culture with GCs, particularly SFGCs, can improve oocyte cytoplasmic quality by reducing ROS levels.

### Granulosa Cells Co-culture Improved Cumulus Expansion of Cumulus–Oocyte Complexes

**FIGURE 5:**
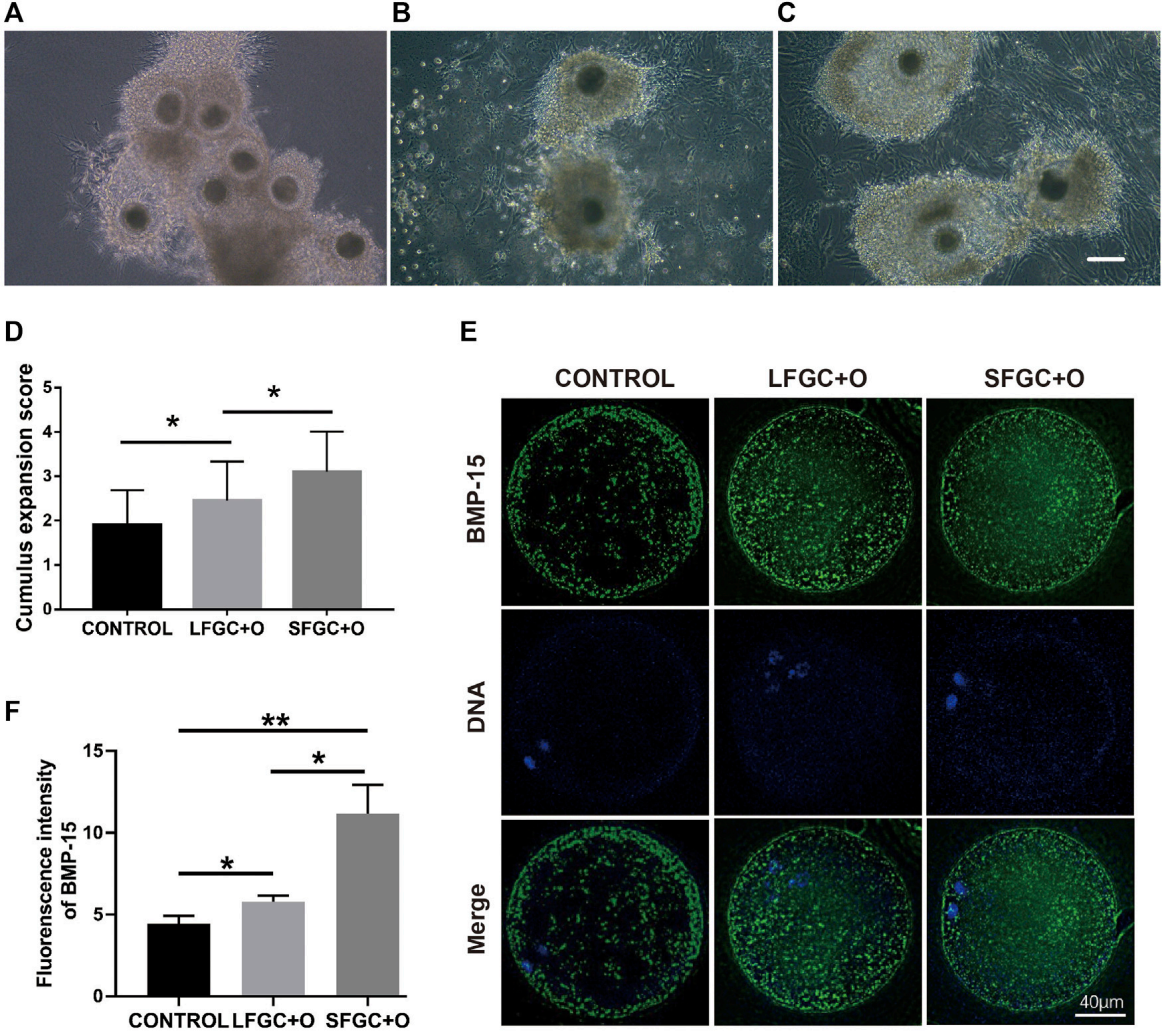
Effects of GC co-culture on cumulus cell expansion and BMP15 expression in mare MII oocytes. Morphology of COCs matured without GCs [CONTROL, **(A)**] or with LFGCs [LFGC + O, **(B)**], or SFGCs [SFGC + O, **(C)**] for 36 h was examined. **(D)** The degree of cumulus cell expansion was assessed after IVM (20 oocytes per group). Scale bar = 150 μm. **(E)** Immunofluorescence detection of BMP15 in MII oocytes derived from the CONTROL, LFGC + O, and SFGC + O groups (30 oocytes per group). **(F)** Average optical intensity was measured using ImageJ. Values are presented as means ± SEM. *p < 0.05, **p < 0.01.

To ascertain the affinity of GCs for mare COCs, cumulus cell expansion was measured (Figures 5A–C). Significantly more cumulus cells were found in the GC co-culture groups than in the control group (Figure 5D). To determine the mechanism by which granulosa cells influence cumulus cell expansion, the expression of bone morphogenetic protein 15 (BMP15) in MII oocytes from each group was analyzed. BMP15, a stimulator of cumulus cell expansion, was identified in all the cytoplasmic areas of oocytes matured *in vitro*. In all groups, BMP15 was highly expressed in the peripheral region of oocytes beneath the oolemma (Figure 5E). However, the relative expression of BMP15 was significantly higher in oocytes from the SFGC + O group than in the control group (*p* < 0.01) and LFGC + O group (*p* < 0.05) (Figure 5F), consistent with the results for cumulus cell expansion (Figure 5D).

### Collagen-Associated Genes Affected Cytoplasmic Maturation in Mare Oocytes

**FIGURE 6:**
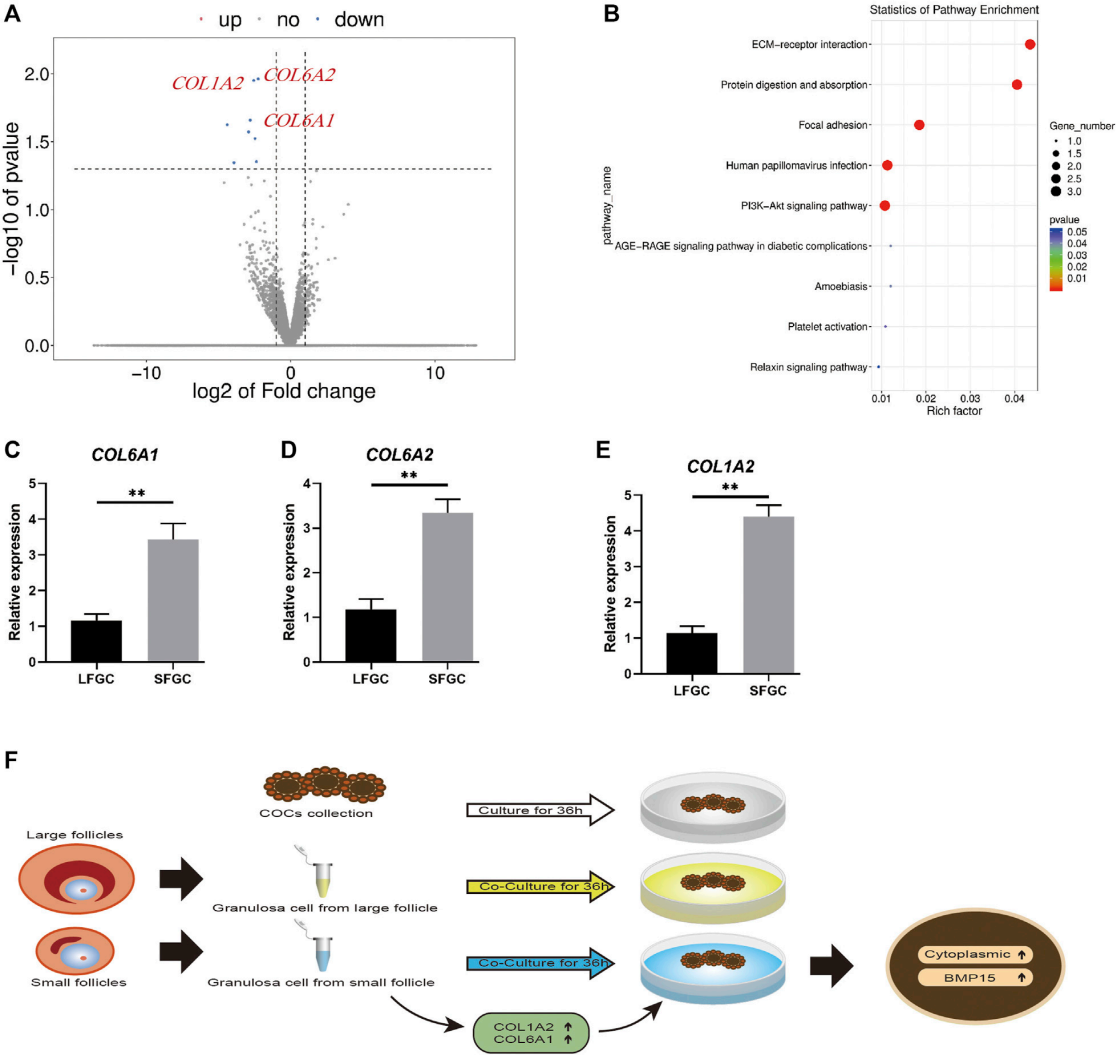
mRNA expression profiles of granulosa cells and validation of differentially expressed mRNAs between granulosa cells from small and large follicles. **(A)** Volcano map displaying DEGs between the granulosa cells from small follicles and those from large follicles. **(B)** KEGG pathway analysis of common DEGs. **(C–E)** Relative expression levels of *COL1A2*, *COL6A1*, and *COL6A2* in large and small follicle–derived granulosa cells were evaluated using q-PCR. **(F)** Speculative model for the beneficial effect of GCs on the cytoplasmic maturation of mare oocytes *in vitro*.

During the *in vitro* maturation stage, there was no significant difference in the mare oocyte nuclear maturation with respect to GCs, although co-culture with GCs, particularly those generated from tiny follicles, could increase the mare oocyte cytoplasmic quality. To learn more about the underlying molecular mechanisms, the transcriptomes of GCs from large and small follicles were sequenced. A volcano plot was constructed to show the top eight DEGs (FC ≥ 2 or FC ≤ 0.5 and *p* < 0.05) (Figure 6A). *COL1A2*, *COL6A1*, and *COL6A2* were enriched in the extracellular matrix (ECM) protein-receptor interaction pathway (Figure 6B), and their expression levels were significantly higher in GCs derived from small follicles than in those from large follicles (Figures 6C–E). The expression patterns of seven DEGs were further confirmed by q-PCR (Figures 6C–E; Supplementary Figure S1). *COL1A2*, *COL6A1*, and *COL6A2* are involved in the regulation of collagens, which might be a key mechanism by which SFGCs improve the cytoplasmic quality of mare oocytes (Figure 6F).

## Discussion

The quality of oocytes is critical for later embryonic development, and growth factors secreted by GCs are important nutrients for the oocyte maturation *in vivo*, although they are lacking in oocyte *in vitro* maturation systems (De Prada et al., 2009). Despite extensive research demonstrating that co-culture with GCs can enhance the maturation efficiency and developmental competence of oocytes in sheep, pigs, humans, and buffaloes (Casillas et al., 2014; Adeldust et al., 2015; Jahromi et al., 2015; Zhang et al., 2022), few reports have described the effects of co-culture with GCs on the IVM of mare oocytes. This is the first study of the effects of GCs on the mare oocyte IVM, revealing that though GCs have no effect on oocyte nuclear IVM efficiency, they substantially increase oocyte cytoplasmic maturity.

Cytoplasmic maturation is essential for mammalian oocyte quality and subsequent embryonic development (Watson, 2007; Mao et al., 2014). One important criterion for oocyte cytoplasmic maturity is the distribution of cortical granules at the periphery of the cell, which is critical for the post-fertilization cortical response initiation to prevent polyspermy (Carneiro et al., 2002; Cheeseman et al., 2016). Mitochondrial redistribution, involving migration toward the central zone after maturation and the homogeneous localization of mitochondria, is considered a marker of cytoplasmic maturation, while a marginal localization is more common in meiotically dysfunctional oocytes (Kirillova et al., 2021). Additionally, mammalian oocytes and embryos are extremely susceptible to oxidative stress; elevated ROS levels will affect the oocyte maturation and embryonic development (Ramalho-Santos et al., 2009). Taken together, the distribution of cortical granules and mitochondria as well as the quantity of ROS are the critical indicators of oocyte cytoplasmic maturity, which were enhanced by co-culture with GCs, particularly those produced from small follicles.

Significantly more enlarged cumulus cells were found in the GC co-culture groups than in the control group, which may be explained by the increased *BMP15* expression in oocytes. When BMP15 was added to the IVM medium, it also had a favorable effect on cumulus expansion in bovines (Caixeta et al., 2013; Delgado et al., 2021). Moreover, null mutations in *BMP15* in oocytes restrict follicular growth, indicating that paracrine factors influence cell viability, cumulus cell expansion, and metabolism during folliculogenesis (Galloway et al., 2000; Su et al., 2008). According to previous research, BMP15 produced by oocytes interacts with BMPR2 on cumulus cells, which is required for the BMP15 type II receptor to convey oocyte signals to cumulus cells for the control of COC growth and apoptosis (Hussein et al., 2006; Liu et al., 2018).

With respect to the molecular mechanism, we found that the expression levels of *COL1A2*, *COL6A1*, and *COL6A2* were significantly higher in GCs derived from small follicles than in those from large follicles, indicating that collagen might be the key factor for improving the mare oocyte IVM. Type I collagen encoded by the *COL1A1* and *COL1A2* genes as well as Type VI collagen encoded by *COL6A1* and *COL6A2* are prominent components of the ECM and are found throughout the ovary (Iwahashi et al., 2000; Berkholtz et al., 2006). The positive effect of collagen on the porcine oocyte IVM maturation has also been observed by using an agarose matrix with ECM proteins, including collagen (Park et al., 2021). Another report has indicated that embedding COCs in collagen gel enhances the meiotic competence of canine oocytes (Otoi et al., 2006). Additionally, collagens were employed to mimic the organ niche of ovarian follicles by enhancing the ECM (Joo et al., 2016). Taken together, our results provide the first evidence for the role of GC co-culture in mare oocyte IVM and suggest that collagens are candidate factors for improving mare oocyte cytoplasmic maturation *in vitro*.

## Data Availability

The raw data supporting the conclusion of this article will be made available by the authors, without undue reservation.
