# Supplementary material for: Granulosa Cells Improved Mare Oocyte Cytoplasmic Maturation by Providing Collagens
Source: Front Cell Dev Biol. 2022 Jun 30;10:914735. doi: 10.3389/fcell.2022.914735 (PMC9280134; doi:10.3389/fcell.2022.914735)
Supplement: Supplementary file 1 [file Table1.DOCX]

**SUPPLYMENTARY DATA**

**TABLE S1. Primers used for real-time polymerase chain reaction analysis.**

| Gene | Forward primer (5’-3’) | Reverse primer (5’-3’) | Annealing temperature (℃) | Fragment size (bp) |
| --- | --- | --- | --- | --- |
| *COL1A2* | ACTGCTGGTCCATCTGGTCCTAG | CATCTCTGCCTGGGTTACCGATTTC | 60 | 124 |
| *COL6A1* | GCCAGACCATCGACACCATCAC | CTTCCCTCGTTCTCCCTCATACCC | 60 | 134 |
| *COL6A2* | TGTTCCACGAGAGGCACGAGAG | GGTCGGAGAAGAGCGTCATGTTG | 60 | 90 |
| *BGN* | CATGCTGAATGACGAGGAGGCTTC | TGAAGGTGGGTGTGAGGGAGTC | 60 | 80 |
| *COCH* | TTCTAATTGACGGCTCCAGCAGTG | TACAGCAGCTATCTTGGCACCAATG | 60 | 119 |
| *GREM1* | CCTTTCAGTCCTGCTCCTTCTGC | TGGTGGGTGGCTGTAGTTCTGG | 60 | 87 |
| *MGP* | GCCTGCTCCTTCTCTCAATTCTGG | CCATCTCTGCTGCGGAGTCATAAAG | 60 | 143 |
| *ENS* | CGTGGTCAAGGTCTTCGCTCTG | GACTCCATCTGGCTTCTGCTTCTG | 60 | 109 |

**TABLE S2. Effect of co-culturing with GCs on mare oocyte development in vitro.**

| Group | No. oocytes | Replications | No. of GV (%) | No. of GVBD (%) |
| --- | --- | --- | --- | --- |
| CONTROL | 30 | 3 | 22 (73.33±6.667) | 8 (26.67±6.667) |
| LFGC+O | 30 | 3 | 23 (76.67±6.667) | 7 (23.33±6.667) |
| SFGC+O | 30 | 3 | 25 (83.33±3.333) | 5 (16.67±3.333) |

Note: Data are the Mean±SEM. LFGC+O: large follicle granulosa cells co-culturing with COCs; SFGC+O: small follicle granulosa cells co-culturing with COCs.

**TABLE S3. Effect of co-culturing with GCs on mare oocyte maturation *in vitro*.**

| Group | Replications | No. of oocytes | Metaphase II (%) |
| --- | --- | --- | --- |
| CONTROL | 6 | 76 | 28 (38.83±3.877) |
| LFGC+O | 6 | 76 | 30 (40±3.497) |
| SFGC+O | 6 | 76 | 37 (49±2.337) |

Note: Data are the Mean±SEM. LFGC+O: large follicle granulosa cells co-culturing with COCs; SFGC+O: small follicle granulosa cells co-culturing with COCs.

**TABLE S4. Effect of GCs co-culturing to cortical granule distribution of mare MII oocyte.**

| Group | Replications | No. of oocyte | Peripheral (%) | Cortical (%) | Homogeneous (%) | Abnormal (%) |
| --- | --- | --- | --- | --- | --- | --- |
| CONTROL |  | 59 | 4 (6.77±0.12) ^c^ | 29 (50.5±8.28) | 21 (33.77±7.41) | 5 (8.97±3.75) |
| LFGC+O |  | 59 | 5 (7.9±2.08) ^b^ | 31 (52.76±6.76) | 21 (35.93±3.57) | 2 (3.4±3.35) |
| SFGC+O |  | 59 | 15 (27.93±0.93) ^a^ | 30 (51.66±7.26) | 13 (18.16±1.45) | 1 (2.23±3.87) |

Note: Data are shown as the Mean±SEM. LFGC+O: large follicle granulosa cells co-culturing with COCs; SFGC+O: small follicle granulosa cells co-culturing with COCs. ^a,b^Within a column, means with a common superscript differed (p < 0.05). ^a,c^Within a column, means with a common superscript differed (p < 0.01).

**TABLE S5. Distribution pattern of mitochondria in mare oocytes in the three groups.**

| Group | Replications | No. of oocytes | Homogeneous (%) | Perpheral (%) | Atrophic (%) |
| --- | --- | --- | --- | --- | --- |
| CONTROL | 3 | 76 | 33 (42.83±4.96) ^c,b^ | 30 (40±2.89)^a^ | 13 (16.67±5.05) |
| LFGC+O | 3 | 76 | 38 (49.5±3.59) ^b^ | 30 (40±2.89)^a^ | 8 (10.5±1.67) |
| SFGC+O | 3 | 76 | 51 (67.67±2.59) ^a^ | 11 (14±1.92)^c^ | 14 (18.33±2.55) |

Note: Data are shown as the Mean±SEM. LFGC+O: large follicle granulosa cells co-culturing with COCs; SFGC+O: small follicle granulosa cells co-culturing with COCs. ^a,b^Within a column, means with a common superscript differed (p < 0.05). ^a,c^Within a column, means with a common superscript differed (p < 0.01).
