# Supplementary figures and images for: Granulosa Cells Improved Mare Oocyte Cytoplasmic Maturation by Providing Collagens
Source: Front Cell Dev Biol. 2022 Jun 30;10:914735. doi: 10.3389/fcell.2022.914735 (PMC9280134; doi:10.3389/fcell.2022.914735)

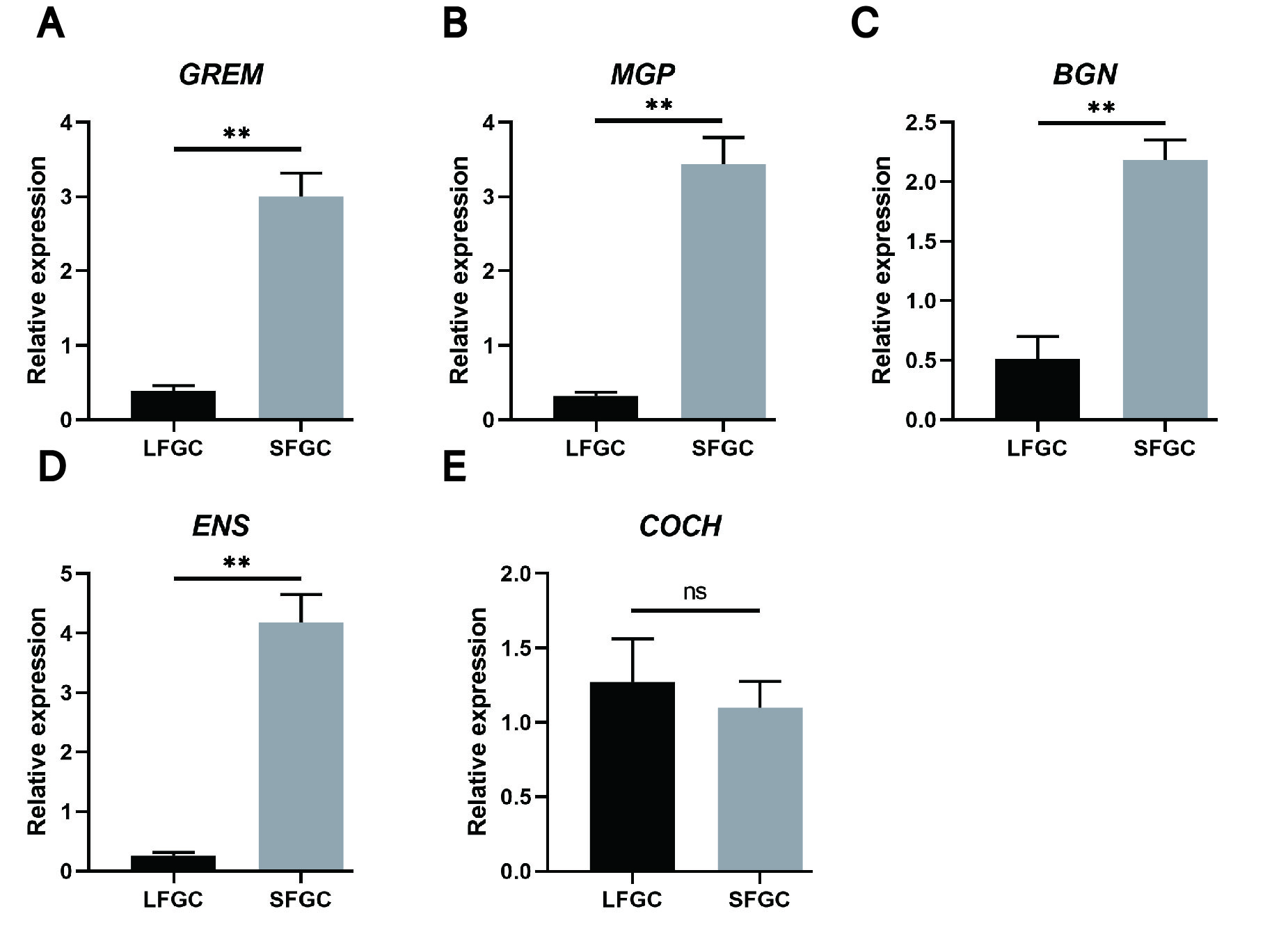

Supplement: Supplementary file 2 [file Image1.JPEG]
